# Dyadic Approaches to the Study of Adult Development and Aging: Emotion Regulation, Attitudes, and Well-Being

**DOI:** 10.1093/geroni/igaf122.1393

**Published:** 2025-12-31

**Authors:** Gloria Luong

## Abstract

Dyadic approaches to the study of aging provide unique opportunities to examine interpersonal processes that may shape psychological development. These four presentations include complementary methodological approaches to some of the latest advances in dyadic processes and adult psychological development. First, English and colleagues investigated interpersonal emotion regulation (IER), whereby regulators attempt to influence the emotions of a target person across two studies including daily diary and lab methods. Greater IER was associated with better emotional and relational well-being, and such effects were more pronounced for older people. Next, Luong examined how married heterosexual couples navigate the transition into older adult housing facilities together. Husbands tended to show greater positive affect and lower depressive symptoms than wives, but they also exhibited lower empathic perspective taking across the 3-5 year longitudinal study. Using three large international panel study datasets, Yang and colleagues demonstrate that romantic couples with different political orientations tend to show a convergence effect over time, such that their political views become closer aligned with their partner’s views. Partners who changed their views and/or showed more moderate political views to begin with, were more likely to have partners who changed their views in line with theirs. Finally, Chopik and colleagues used novel methodological techniques to understand how couples “match” on their love languages, and the implications that matching may have on relationship success. These talks highlight advantages to dyadic approaches to studying aging for both individuals and couples. Dyadic Health Research Interest Group Sponsored Symposium

